# From Teeth to Therapy: A Review of Therapeutic Potential within the Secretome of Stem Cells from Human Exfoliated Deciduous Teeth

**DOI:** 10.3390/ijms241411763

**Published:** 2023-07-21

**Authors:** Nurul Hafizah Mohd Nor, Nur Izzati Mansor, Mohd Izhar Ariff Mohd Kashim, Mohd Helmy Mokhtar, Farah Ayuni Mohd Hatta

**Affiliations:** 1 Institute of Islamic Civilization, Universiti Kebangsaan Malaysia, Bangi 43600, Selangor Darul Ehsan, Malaysia; nurulhafizah@ukm.edu.my (N.H.M.N.); izhar@ukm.edu.my (M.I.A.M.K.); farahayuni@ukm.edu.my (F.A.M.H.); 2 Department of Nursing, Faculty of Medicine, Universiti Kebangsaan Malaysia, Cheras 56000, Kuala Lumpur, Malaysia; 3 Faculty of Islamic Studies, Universiti Kebangsaan Malaysia, Bangi 43600, Selangor Darul Ehsan, Malaysia; 4 Department of Physiology, Faculty of Medicine, Universiti Kebangsaan Malaysia, Cheras 56000, Kuala Lumpur, Malaysia; helmy@ukm.edu.my

**Keywords:** secretome, conditioned medium, SHED, dental stem cells, tissue regeneration

## Abstract

Stem cells derived from human exfoliated deciduous teeth (SHED) have emerged as an alternative stem cell source for cell therapy and regenerative medicine because they are readily available, pose fewer ethical concerns, and have low immunogenicity and tumourigenicity. SHED offer a number of advantages over other dental stem cells, including a high proliferation rate with the potential to differentiate into multiple developmental lineages. The therapeutic effects of SHED are mediated by multiple mechanisms, including immunomodulation, angiogenesis, neurogenesis, osteogenesis, and adipogenesis. In recent years, there is ample evidence that the mechanism of action of SHED is mainly due to its paracrine action, releasing a wide range of soluble factors such as cytokines, chemokines, and trophic factors (also known as ‘secretome’) into the local tissue microenvironment to promote tissue survival and recovery. This review provides an overview of the secretome derived from SHED and highlights the bioactive molecules involved in tissue regeneration and their potential applications in regenerative medicine.

## 1. Introduction

In this review, we provide an overview of the literature on the secretome derived from SHED, focusing on their secreted soluble factors and therapeutic potential in regenerative medicine. We conducted an extensive search of the scientific literature for secretome derived from SHED, with a specific emphasis on therapeutic potential. The search terms “stem cells from human exfoliated deciduous teeth”, “secretome”, and “conditioned medium” were used to find articles in four academic search engines: PubMed, Google Scholar, Science Direct, and Medline. Original research articles in which any type of testing procedure was used were included in the search. Studies involving humans and animals were also included. Data available in English were included regardless of the year of publication.

### 1.1. Overview of Mesenchymal Stem Cells

Mesenchymal stem cells (MSCs) are multipotent stem cells, capable of self-renewal, and have the potential to differentiate into tissues of multiple lineages [1,2,3,4]. MSCs are found in almost all tissues of the body, including bone marrow [5,6,7], adipose tissue [8,9], umbilical cord [10,11,12], and amniotic fluid [13,14,15,16]. MSCs consist of a heterogeneous population of multipotent cells [17]. MSCs are characterised by their expression of cell surface markers such as CD73, CD90, and CD105, while they lack expression of CD45, CD34, CD11b, CD14, CD19, CD79a, and the human leukocyte antigen (HLA) class II [1,18].

MSCs are considered promising cell-based therapies for various diseases due to their multilineage potential, immunomodulatory properties, and ability to secrete a broad range of trophic factors and cytokines [3,19]. Bone marrow-derived MSCs (BM-MScs) were the first to be discovered [2,9,20] and are most commonly used in the clinical treatment of stroke, cardiovascular disease, and cartilage injury [21,22,23]. For instance, Gupta et al. (2016) conducted a phase II clinical trial using allogeneic BM-MSCs (Stempeucel^®^) in combination with hyaluronic acid to treat patients with knee osteoarthritis (KOA). Although no significant changes were observed on MRI analysis, the study showed promising therapeutic potential of MSCs in relieving pain and modulating the functional status of the knee joint [21]. In addition, BM-MSCs have been investigated for their therapeutic potential in treating neurological disorders, including ischemic stroke. In the phase I/II clinical trial, Levy et al. (2019) revealed the safety of intravenous administration of allogeneic BM-MSCs and observed improved functional recovery in patients with chronic ischemic stroke [22]. Law et al. (2021) found similar findings, demonstrating the therapeutic effects of autologous BM-MSCs in patients with subacute middle cerebral artery (MCA) infarction [23].

Since the isolation of MSCs from bone marrow is highly invasive, time-consuming, and insufficient, the umbilical cord has emerged as an alternative MSCs source for cell-based therapy. In recent years, stem cells from various dental tissues have been established by several scientists as novel and promising candidates for cell-based therapy and regenerative medicine. Dental stem cells have been extensively explored in dental diseases and are effective for other diseases [24,25,26]. In this context, the following sections describe detailed information about the types and origins of dental stem cells and their properties.

### 1.2. Human Dental Stem Cells

**Figure 1:**
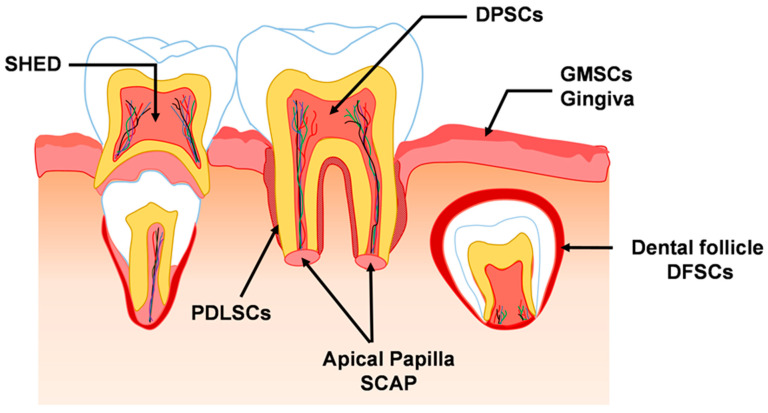
A schematic illustration of the origin and the various subpopulations of dental stem cells (DSCs). DPSCs: dental pulp stem cells; PDLSCs: periodontal ligament stem cells; SCAP: stem cells from apical papilla; SHED: stem cells from human exfoliated deciduous teeth; DFSCs: dental follicle stem cells; GMSCs: gingival mesenchymal stem cells.

In addition to bone marrow, adipose tissue, and umbilical cord, MSCs can also be obtained and isolated from exfoliated deciduous teeth and extracted premolars and third molars. These tissues are often discarded as biological waste, and the process of deriving stem cells from these tissues is noninvasive [27] and less ethically questionable [28]. The derived cells have been shown to share common characteristics with other mesenchymal stem cells [29,30]. To date, several subpopulations of dental-derived stem cells have been identified, including dental pulp stem cells (DPSCs), periodontal ligament stem cells (PDLSCs), stem cells from apical papilla (SCAP), stem cells from human exfoliated deciduous teeth (SHED), dental follicle stem cells (DFSCs), and gingival mesenchymal stem cells (GMSCs). In the permanent tooth, DPSCs are found in the pulp tissue of the extracted tooth [31], PDLSCs are isolated from the periodontal ligament, which is a specialised cellular connective tissue between the alveolar bone and cementum of the extracted tooth [32,33], and SCAP are derived from papillary tissue in the apical root of the developing tooth [34,35]. In contrast to DPSCs, SHED are derived from the dental pulp of an exfoliated deciduous tooth [36]. DFSCs are isolated from the dental follicle tissue surrounding the papilla and enamel of the developing tooth, and GMSCs are found in the lamina propria of the gingival tissue [37,38]. Figure 1 illustrates the origin and various subpopulations of dental stem cells.

The characterisation and differentiation potential of human dental-derived stem cells are summarised in Table 1. Most researchers reported that the MSC surface markers expressed in bone marrow and umbilical cord MSCs, including CD44, CD73, CD90, and CD105, are also expressed in all subpopulations of dental stem cells. Moreover, dental stem cells do not express haematopoietic and lymphocytic markers such as CD14, CD34, CD45, and human leukocyte antigen-DR (HLA-DR; <2%). Other surface markers reported in previous work include CD10, CD13, CD29, CD44, and CD59 [39,40,41,42]. The dental-derived stem cells also expressed Stro-1, a marker for mesenchymal progenitor cells [43,44]. Interestingly, the majority of dental-derived stem cell subpopulations exhibited the expression of key pluripotency markers, including Nanog, Oct4, Sox-2, SSEA-3, and SSEA-4. This suggests their potential to differentiate into multiple developmental lineages [39,40,41,42], despite the relatively low expression levels of these markers.

As far as hormesis is concerned, human dental stem cells are susceptible to low-intensity stressors or stimuli such as radiation or oxidative stress, which can cause the activation of specific signalling pathways involved in tissue regeneration. As a result of this activation, there is an increase in the production of growth factors and cytokines, which are essential for promoting the proliferation and differentiation of dental stem cells. This thus increases the ability of dental stem cells to regenerate tissue. Hence, it could be suggested that hormesis may be an important factor in the maintenance and healing of dental tissue, thus opening a potential avenue for a therapeutic approach. In light of this, utilising the idea of hormesis may transform regenerative dentistry by increasing dental stem cells’ inherent tissue regeneration capabilities.

### 1.3. Overview of MSC-Derived Secretome

Growing evidence indicates that the therapeutic benefits of MSCs primarily result from paracrine actions of its soluble factors such as cytokines, chemokines, trophic factors, and extracellular vesicles (EVs), which are collectively termed as ‘secretome’. These soluble factors exhibit considerable potential in promoting endogenous tissue repair and regeneration [50,51,52]. Moreover, the potential risks associated with MSC-based treatments, such as thromboembolism and fibrosis, have sparked interest in exploring the therapeutic potential of MSC-derived secretome as an alternative approach.

Numerous investigations have been carried out to examine the composition and properties of the secretome released by MSCs. The composition of MSC-derived secretome varies based on the source of MSCs and microenvironmental stimuli, i.e., hypoxia and inflammation [52,53,54]. The resultant secretome plays a crucial role in maintaining cellular homeostasis and facilitating tissue regeneration. A study conducted by Bartaula-Brevik et al. (2017) revealed that a hypoxic microenvironment influenced the expression of inflammatory cytokines (IL-1β, IL-6, and IL-8) and VEGF-A in the MSC-derived secretome when compared to normoxic conditions [53]. This finding suggests that hypoxia modulates the composition of the MSC-derived secretome, thereby contributing to enhanced angiogenesis and wound healing.

Another study by Shin et al. (2021) investigated the secretome profiles of four different human MSC sources: BM-MSCs, adipose tissue (AD-MSCs), placenta (PL-MSCs), and Wharton’s jelly (WJ-MSCs). The study identified 265 proteins in AD-MSCs, 253 proteins in BM-MSCs, 511 proteins in PL-MSCs, and 440 proteins in WJ-MSCs. These proteins were found to have remarkable roles in various biological functions, such as cellular development, migration and metabolic activities. The authors further emphasised that despite the distinct origins of the MSCs and microenvironmental stimuli, their secretomes exhibited similar functional properties [50]. Therefore, these findings offer valuable insights that highlight the potential applications of MSC-derived secretomes in the fields of regenerative medicine and tissue engineering.

## 2. Stem Cells from Human Exfoliated Deciduous Teeth (SHED)

Deciduous teeth, also known as primary teeth, baby teeth, or milk teeth, are the first set of teeth to develop in humans. Deciduous teeth differ from permanent teeth, also known as second teeth, in their anatomy, function, and stage of development [43]. During the transition from childhood to adulthood, the permanent teeth erupt at the same time as the roots of the deciduous teeth, which recede to make room for the permanent teeth [43]. On average, humans have 20 deciduous teeth, and it takes more than seven years for all of these teeth to be replaced by permanent teeth [55].

The deciduous teeth already contain dental pulp prior to birth, which remains until the permanent teeth erupt [56]. As this dental pulp is already present during prenatal development, it represents a dynamic niche containing stem cells that have not yet been significantly influenced by their genetic and/or environmental context [19,56]. Human dental pulp stem cells are neurotrophic and originate from the embryonic neural crest, which distinguishes them from mesenchymal stem cells from other tissues [57]. Exfoliation of healthy deciduous teeth can result in the recovery of a type of dental stem cell known as SHED. As decaying deciduous teeth are considered waste and are usually discarded, the harvesting of these stem cells is less invasive and does not cause morbidity [58]. This makes them a readily available tissue source with the potential to provide sufficient cells for various clinical applications.

SHED are a type of postnatal stem cell [43]. They are a population of immature stem cells [58], specifically immature DPSCs [59]. In addition, they may be even more immature than other postnatal stromal stem cell groups studied. In vitro, SHED appear to be flattened, elongated, large, or fibroblast-like shape.

Although SHED have embryonic stem cell markers, they are also counted as MSCs because these ubiquitous cells have specific markers normally found only in MSCs and other pluripotent stem cells, namely Nanog, CD73, STRO-1, STRO-1, CD166, CD146, SOX2, CD105, and SSEA4, but are negative for CD34 and CD45 [39]. As a type of postnatal stem cell associated with the neural crest, these cells have been shown to possess a number of neuronal cell markers, notably Nestin, NFM, GAD, NeuN, CNPase, GFAP, and β-III tubulin [39].

SHED are able to maintain their ability to remain in an undifferentiated state without loss of cell viability and remain stable [56,58,60,61], even when cryopreserved for a long period of time. The ability to differentiate into different cell types, such as fibroblasts [58], epithelial cells [61], odontoblasts, adipocytes, neuronal cells, and so forth, makes SHED a promising cell source for regenerative medicine. In addition, SHED raise fewer ethical and legal concerns compared to other potential sources of dental stem cells.

## 3. Secretome Derived from SHED

The secretome could be described as a conditioned medium (CM) in combination with an EV fraction derived from cultured stem cells [62]. The secretome contains different profiles and properties of cytokines and growth factors depending on the cell sources, such as mesenchymal stem cells, umbilical cord stem cells, dental stem cells, and many others. Among the cell sources available, the study of the secretome isolated from dental stem cells, particularly SHED, has so far not yet received wide attention.

Previous studies have shown that the secretome of SHED contains a variety of cytokines, paracrine factors, and growth factors. According to the cytokine membrane array analysis by Kang et al. (2022), SHED expressed various cytokines at different levels, including ANGPT2, AgRP, Axl, AREG, BTC, BMP4, bFGF, BMP6, IGFBP1, IGFBP2, CXCL1, CXCL5 CXCL11, CCL3, CCL4, CCL23, CCL25, CNTF, CTACK, VEGF, VEGF-D, EGRF, EGF, FGF-4, FGF-9, GITR, GM-CSF, PLGF, ICAM-1, ICAM-3, IGF-1, IGF-1 sR, IGFBP3, IGFBP6, IL-1 R1, IL-1 ra, IL-2, IL-2R alpha, IL-12 p70, IL-3, IL-4, IL-5, IL-6, IL-7, IL-11, IL-17, LIGHT, MDC, MIF, MIG, MCP-1, MSP alpha, MIP-3 beta, NT-3, NT-4, GCP-2, BDNF, CXCL13 (BLC), CX3CL1, PARC, THPO, TRAIL R3, XCL1, Eotaxin1, IFN-gamma, and β-NGF [63]. Additionally, Hiraki et al. (2020) detected ANG, bNGF, BDNF, BMP-2, BMP-4, EGF, TIMP-1, IL-6, FGF-2, GDNF, HGF, MCP-1, OPG, M-CSF, OPN, NT-3, PDGF-BB, TIMP-1, VEGF-A, and VEGF-C in SHED-derived secretome, although they were unable to detect IGFBP3 [64].

In comparison with secretomes of other mesenchymal stem cells, a prior study by Konala et al. (2020) showed that SHED-derived secretome was superior to BM-MSCs in reducing scarring, angiogenesis, inflammation, fibrosis, and immunomodulation [65]. The study, which also analysed the secretomes of SHED, BM-MSCs, and WJ-MSCs, revealed that SHED-derived secretome has higher expression levels of (ANG)1, PDGF, IL-10, SDF-1, HGF, (VEGF)1, FGF-2, and IFN-γ than those derived from BM-MSCs and WJ-MSCs. Additionally, it was found that SHED-derived secretome has more IL-6 than that in BM-MSCs.

Moreover, since SHED are suggested to be a more potentially useful source of stem cells than BM-MSCs and DPSCs in cell therapy [66], therefore it could be suggested that the secretome derived from SHED contains a wider range of bioactive molecules that could enhance tissue regeneration and repair and hence, considered as a suitable candidate for a cell-free approach in regenerative medicine and dentistry. However, further research is needed to fully understand the mechanisms underlying the therapeutic effects of the SHED-derived secretome and to optimise its clinical application in future.

## 4. Secretome Derived from SHED in Tissue Regeneration: Evidence from Experimental Studies

The secretome derived from SHED or preferably called SHED-CM in a number of studies, can stimulate various biological activities in different cell types in vitro as well as in vivo through complicated processes. In most studies, the SHED-CM was prepared by collecting the medium from SHED cultured for at least 48 h in serum-free supplemented media, usually Dulbecco’s Modified Eagle’s medium (DMEM). The SHED-CM was then centrifuged to remove all cell debris. The supernatant from SHED-CM obtained during centrifugation was usually used without enrichment or dilution for subsequent experimental analyses.

Studies have shown that the secretome derived from SHED holds great potential as a therapeutic agent for various diseases and injuries related to tissue regeneration and has been shown to play significant roles in proliferation, apoptosis, angiogenesis, osteogenesis, chondrogenesis, immunomodulation, and immunoregulation in many pathological conditions.

### 4.1. Effect of SHED-Derived Secretome on Proliferation and Apoptosis

Much evidence has revealed the remarkable ability of SHED-derived secretome to promote proliferation and inhibit apoptosis. In a mouse model of perinatal hypoxia–ischemia (HI)-induced brain injury, SHED-CM exhibited significant reductions in apoptosis and tissue loss [25]. Additionally, in a mouse model of streptozocin-induced diabetes, SHED-CM has been shown to promote the proliferation of pancreatic β-cells and enhance insulin secretion [67].

Yamaguchi et al. (2015) showed that SHED-CM exhibited a significant suppressive effect on apoptosis in a mouse model of ischemic heart, as evidenced by a remarkable reduction in TUNEL-positive cardiomyocytes compared to untreated mice [68]. Matsushita et al. (2017) discovered that SHED-CM secreted various tissue-regenerating factors known for their roles in antiapoptosis and hepatocyte protection, as well as in the proliferation and differentiation of liver progenitor cells [69].

### 4.2. Effect of SHED-Derived Secretome on Immunomodulation and Immunoregulation

Several studies revealed that the secretome derived from SHED has a therapeutic effect through its immunomodulatory and anti-inflammatory actions. Matsubara et al. (2015) discovered that SHED-CM exhibited immunomodulatory and anti-inflammatory effects characterised by a reduction in proinflammatory cytokine levels and induction of M2 anti-inflammatory macrophages in a rat model of spinal cord injury (SCI) [70]. These effects led to a remarkable recovery of hindlimb locomotor function. Wakayama et al. (2015) demonstrated that SHED-CM significantly suppressed the mRNA expressions of TNF-α, IL-6, IL-1β, and iNOS while simultaneously upregulating the expressions of M2 cell markers in bleomycin (BLM)-induced acute lung injury (ALI) in mice [71]. The study also confirmed the ability of SHED-CM to induce M2 differentiation in bone marrow-derived macrophages through in vitro experiments.

Similar findings were also reported by other studies, in which SHED-derived secretome exhibited a potent anti-inflammatory effect in both a rat model of acute liver failure (ALF) [69] and a mouse model of ischemia-reperfusion (I/R) [68]. In a mouse model of autoimmune encephalomyelitis, SHED-CM demonstrated its ability to suppress proinflammatory cytokine levels, inhibit inflammatory cell infiltration, and reduce demyelination in the spinal cord. Moreover, SHED-CM effectively inhibited the proliferation of myelin oligodendrocyte glycoprotein-specific CD4^+^ T cells, which played a crucial role in the progression of autoimmune encephalomyelitis disease [72].

### 4.3. Effect of SHED-Derived Secretome on Angiogenesis

Angiogenesis plays a pivotal role in facilitating tissue regeneration. Research on the angiogenesis capacity of SHED-derived secretome has been conducted in various pathological conditions. Sugimura-Wakayama et al. (2015) demonstrated the capability of SHED-CM to promote tube formation in HUVECs and induce the expression of angiogenic factor VEGF in Schwann cells in vitro [73]. Similarly, de Cara et al. (2019) demonstrated the angiogenic effects of SHED-CM in HUVECs [74]. The study also revealed that 30-day treatment with SHED-CM resulted in the successful induction of vascularised connective tissue within the root canal in a rat orthotopic model of dental pulp regeneration. 

Kato et al. (2020) revealed that SHED-CM not only promoted tube formation in HUVECs but also accelerated HUVECs migration in wound healing and Boyden chamber assays [75]. SHED-CM was also able to promote ex vivo neovascularisation, as evidenced by the formation of neovessel sprouting from the aortic rings of Sprague Dawley rats. The in vivo study by Hiraki et al. (2020) also proposed cytokines and growth factors in SHED-derived secretome, particularly M-CSF, ANG, VEGF-A, MCP-1, VEGF-C, and bFGF, to be the main actors in the angiogenesis in the calvarial bone defect model of a mouse [64]. Additionally, VEGF, together with HGF, were also found to play roles in angiogenesis stimulation in the pressure ulcer mouse model [76].

### 4.4. Effect of SHED-Derived Secretome on Osteogenesis and Chondrogenesis

Previous studies reported that secretome derived from SHED plays important roles in promoting osteogenesis and chondrogenesis. Hiraki et al. (2019) reported that SHED-CM enhanced bone regeneration in a mouse model of the calvarial bone defect model [64]. This was evidenced by the increased formation of mature bone, increased abundance of collagen fibres and osteiods, and was notably superior to untreated mice. The study further revealed that SHED-CM contained a rich concentration of bone-metabolism-related markers, including OPG, OPN, BMP-2, and BMP-4, which likely contributed to bone regeneration.

Muhammad et al. (2020) investigated the regenerative effect of SHED-CM on osteoarthritis chondrocytes (OA) in vitro [77]. Interestingly in this study, SHED-CM was first cultured in a serum-free medium for 48 or 72 h before being subjected to the interleukin-1β-stimulated chondrocytes for 24, 48, and 72 h. The growth factors in SHED-CM, such as TGF-β1, IL-10, and IL-6, were involved in chondrocyte growth, viability, proliferation, and protection. The treatment with SHED-CM not only improved the cell viability but also increased the expression of major markers of extracellular articular cartilage, aggrecan, and collagen type 2 (COL 2) in OA chondrocytes. Additionally, SHED-CM exhibited a modulatory effect on OA-induced inflammation by downregulating the levels of MMP-13 and NF-κB in OA chondrocytes. The findings were consistent with another study conducted by Giannasi et al. (2020), which also demonstrated the positive effects of the secretome derived from adipose-derived stem cells (ASCs) in protecting human articular chondrocytes (CH) from OA damage. The study revealed that the ASC-derived secretome reduced MMP activity and the expression of proinflammatory mediators associated with OA [78]. These consistent findings provide additional support for the potential therapeutic benefits of utilizing the secretome as an effective strategy to alleviate OA-induced inflammation and injury.

Vu et al. (2020) reported that SHED-CM treatment significantly restored the odontoblast/osteogenic differentiation of hydrogen peroxide (H_2_O_2_)-induced DPSCs, contributed by the effect of TGF-β, MMP, VEGF, FGF, Ils, and BMP in SHED-CM [62]. This was evidenced by the increased levels of odontoblast/osteogenic-related markers and enhanced mineral deposition in DPSCs, following treatment with SHED-CM. The study also suggested that these biological activities may be related to SMAD protein phosphorylation and MAPK signalling pathway.

### 4.5. Effect of SHED-Derived Secretome on Neuroprotection and Neuroregeneration

A growing body of literature has evaluated the neuroprotection and neuroregenerative effects of SHED-derived secretome in vitro and in vivo. Sugimura-Wakayama et al. (2015) reported various cytokines and growth factors such as NT-3, NGF, CNTF, HGF, GDNF, BDNF, and VEGF in SHED-derived secretome played an additive or synergistic role in peripheral nerve regeneration and in stimulating neuritogenesis, angiogenesis, and migration of Schwann cells, as well as in neurite outgrowth in dorsal root ganglion (DRG) neurons. The regeneration and recovery of nerves and axons were also observed in the in vivo study of the rat sciatic nerve model [73].

The SHED-derived secretome also showed benefits in treating neurodegenerative diseases. In a mouse model of perinatal HI-induced brain injury, treatment with SHED-CM led to a notable improvement in survival rate and neurological functions [25]. A study of permanent middle cerebral artery occlusion (pMCAO) in rats showed that SHED-CM promoted the migration and differentiation of endogenous neuronal progenitor cells (NPCs), reduced infarct volume, stimulated vasculogenesis, and subsequently improved motor function recovery [66].

In the assessment of Alzheimer’s disease (AD) mouse models, Mita et al. (2015) showed that SHED-CM improved cognitive function and attenuated Aβ-induced inflammation, thus protecting the neurons against Aβ toxicity [79]. The study also found that SHED-CM suppressed glutamate-induced neuronal death in primary cerebral neurons derived from mouse embryos.

In the evaluation of the neurogenic potential of SHED-derived secretome for the treatment of Parkinson’s disease (PD), Fujii et al. (2015) reported that CM obtained from both SHED and dopaminergic neuron-like SHED (dSHED) were able to protect the neurons against 6-OHDA toxicity and promoted neurite outgrowth in vitro [80]. Chen et al. (2020) found that SHED-CM improved motor deficits in the rotenone-induced PD rat model, marked by increased expression of tyrosine hydroxylase (TH) in the striatum and decreased in α-synuclein levels in nigra and striatum regions. Moreover, SHED-CM treatment significantly attenuated neuroinflammation in the brain of rotenone-induced PD rats by decreasing the Iba-1 and CD4 levels in the striatum, nigra, and cortex regions [81].

Taken together, these findings revealed the generative properties of SHED-derived secretome and its potential for the treatment of various pathological conditions. Table 2 summarises the potential of the secretome of SHED in tissue regeneration, as reported in previous studies.

## 5. Challenges in the Use of Secretome in Tissue Regeneration

Despite the many potential benefits of SHED-derived secretome, there are still some challenges that need to be addressed. One of these challenges is optimising the production and use of secretome. Currently, there is no standardised protocol for the production of the secretome from SHED, and the methods used can significantly affect the quality and quantity of the secretome obtained. Further research is needed to develop more efficient and consistent methods for the production and use of SHED-derived secretome. Another challenge is the need for a better understanding of the mechanisms behind the properties of SHED-derived secretome. While the effects of SHED-derived secretome have been extensively researched, the specific mechanisms underlying these effects are not yet fully understood. Further research is needed to determine how SHED-derived secretome exerts its immunomodulatory and regenerative effects and to optimise its use in the treatment of various diseases and injuries.

## 6. Ethical Issues in the Use of Secretome

From moral and ethical perspectives, some groups of people have too extreme understanding of religion and reject the concept of modern technology because they believe that this technology wants to change God’s creation on the human body. For this group, modern stem cell technology is a threat to the way of life of future generations because it allows tissue regeneration even for medical purposes. Superficial religious thinking such as this must be set aside because it is built without proper scientific evidence, as has been demonstrated by scholars who are experts in the field of ethics and religion. Therefore, these efforts must be supported to ensure the welfare of human beings in accordance with the passage of time. It should be noted that all of God’s creations should be used for the benefit of man, even if they have to produce a secretome for tissue regeneration.

## 7. Conclusions

SHED have become an increasingly important tool in regenerative medicine. As scientists have gained a better understanding of SHED and their secretome, interest has grown in its potential applications in the treatment of various diseases and injuries. In this study, we have revisited the properties of the SHED-derived secretome, highlighted its unique characteristics and discussed the potential benefits of using the SHED-derived secretome in regenerative medicine. The properties of SHED-derived secretome make it an attractive tool for use in regenerative medicine. Its immunomodulatory effects, its ability to promote tissue regeneration and angiogenesis, and its lower risk of tumour formation and immune rejection make it a promising alternative to live cell therapy. However, further research is needed to understand the mechanisms underlying these properties fully and to optimise their production and use. With further research and development, the secretome of SHED has the potential to become a valuable tool in the field of regenerative medicine.

## Figures and Tables

**Table 1:** Properties of human dental-derived stem cells.

Cell Population	Stem Cell Markers	Immunophenotype	Differentiation Potential	Reference
DPSCs	STRO-1, Nanog, Oct4, Sox-2, SSEA-3, SSEA-4	CD10+, CD13+, CD29+, CD44+, CD59+, CD73+, CD90+, CD105+, CD106+, CD146+, CD14-, CD34-, CD45-, HLA-DR-	Odontogenic, osteogenic, neurogenic, adipogenic, myogenic, chondrogenic	[40,42]
PDLSCs	STRO-1, Nanog, Oct-4, Sox-2, Rex-1, SSEA-1, SSEA-3, SSEA-4, TRA-1-60, TRA-1-81	CD9+, CD10+, CD13+, CD26+, CD29+, CD44+, CD59+, CD73+, CD90+, CD105+, CD106+, CD146+, CD166+, CD14-, CD34-, CD45-, HLA-DR-	Osteogenic, cementogenic, adipogenic, chondrogenic, insulin-producing cells	[41,45]
SCAP	STRO-1, Nanog, Oct4	CD13+, CD24+, CD29+, CD44+, CD73+, CD90+, CD105+, CD106+, CD146+, CD166+	Odontogenic, osteogenic, neurogenic, adipogenic	[40]
SHED	STRO-1, Nanog, Oct4, Nestin, SSEA-3, SSEA-4, TRA-1-60, TRA1-81	CD13+, CD29+, CD44+, CD73+, CD90+, CD105+, CD106+, CD146+, CD166+, CD14-, CD34-, CD45-, HLA-DR-	Odontogenic, osteogenic, neurogenic, adipogenic, myogenic, chondrogenic	[39,46,47]
DFSCs	STRO-1, Nanog, Oct4, SSEA-4	CD9+, CD10+, CD13+, CD29+, CD44+, CD53+, CD59+, CD73+, CD90+, CD105+, CD106+, CD146+, CD166+, CD14-, CD34-, CD45-, HLA-DR-	Odontogenic, osteogenic, neurogenic, adipogenic	[40,48]
GMSCs	STRO-1, Nanog, Oct4	CD13+, CD29+, CD44+, CD73+, CD90+, CD105+, CD146+, CD14-, CD34-, CD45-, HLA-DR-	Osteogenic, neurogenic, adipogenic, chondrogenic, endothelial	[41,49]

**Table 2:** Previous studies on the secretome derived from SHED.

Type of Study	Purpose	Secreted Soluble Factors	Key Findings	References
In vitro (SHED vs DPSCs)	To compare cytokine profiles produced by SHED and DPSCs.	IL-6, CNTF, CCL23, IGFBP2, IL-7, EGF, BMP6, IGFBP1, GM-CSF, Eotaxin1, IL-5, IFN-gamma, PARC, IL-2, BLC, BDNF, MCP-1	SHED-derived secretome expressed more cytokines involved in odontogenesis, osteogenesis, and immunomodulation, while DPSCs-derived secretome expressed more cytokines involved in angiogenesis.	[63]
In vitro (DPSCs)	To investigate the potency of SHED-CM on DPSCs in pulp regeneration.	TGF-β, MMP, VEGF, FGF, Ils, BMP	SHED-CM showed a dose-dependent promotive effect on the proliferation, migration, and survival of DPSCs. Upregulation of marker genes for odontoblasts and osteogenesis and increased mineral deposition of impaired DPSCs in the presence of SHED-CM.	[62]
In vivo (Mouse calvarial bone defect model)	To investigate the effect of SHED-CM on bone regeneration.	TIMP-1, OPG, OPN, M-CSF, MCP-1, HGF, ANG, VEGF-C, IL-6, BDNF, NT-3, BMP-4, BMP-2, bNGF, FGF-2, GDNF, PDGF-BB, EGF	Bone regeneration was improved in the defects treated with stem cells and CM compared to controls 8 weeks after transplantation. Mature bone formation and angiogenesis were confirmed with SHED-CM but not with stem cells or in controls.	[64]
In vitro (OA chondrocytes)	To evaluate the regenerative effect of SHED-CM on OA chondrocytes for cartilage repair and regeneration.	TGF-β1, IL-10, IL-6	SHED-CM protected chondrocytes by increasing matrix proteins and suppressed MMP-13 expression. SHED-CM attenuated the inflammatory assault induced by IL-1β. The regenerative effect of SHED-CM could be attributed to secreted factors modulating catabolic processes towards an anabolic phenotype by downregulating NF-κB.	[77]
In vitro (HUVECs) In vivo (Mouse Matrigel plug assays) Ex vivo (Rat aortic ring assay)	To examine the beneficial effects of secreted factors from SHED on endothelial cells to promote angiogenesis.	n/a	SHED-CM significantly increased the proliferation of HUVECs. SHED-CM accelerated the migration of HUVECs in wound healing and Boyden chamber assays. SHED-CM induced complex tubular structures of HUVECs in a tube formation assay. SHED-CM significantly increased neovascularisation in rat aorta. The angiogenic effects of SHED-CM were equal to or greater than the effective concentration of VEGF.	[75]
In vitro (HUVECs) In vivo (Rat model of orthotopic dental pulp regeneration)	To evaluate the effect of SHED-CM on the proliferation, differentiation, migration ability, cell death, gene expression, and production of VEGF.	n/a	SHED-CM significantly induced lower expression of 7AAD in HUVECs, whereas the expression of the Ki67 was similar in all groups. SHED-CM induced expression of VEGF-A. SHED-CM significantly induced higher VEGF synthesis than other media. SHED-CM induced the formation of vascularised connective tissue inside the root canal.	[74]
In vitro (Human breast cancer stem cells (BCSCs))	To evaluate the stemness and proliferation of human BCSCs after being supplemented with heated secretome from SHED.	n/a	The heated secretome of SHED contained activated TGF-β1, which increased the expression of stemness genes, OCT4, and ALDH1A1, as well as the proliferation of human BCSCs (ALDH+) via TGF-β1 paracrine signalling.	[82]
In vivo (Rat with ALF)	To study the multifaceted therapeutic benefit of SHED-CM in ALF in rats.	HGF, MMP-10, MCP-1, ANG, SCF, IGFBP-2, sIL-6R, EGFR, FSTN, MMP-3, spg130, GRO, MIP-1β, MIF, RAGE, TIMP-4, adipsin, OPG, CXCL16, IGFBP-1, BDNF, LAP, GDNF, sTNFR1, TGF-β2, FGF-7, MMP-13, MMP-9, Flt-3 L, Dkk-3, NID-1, VEGF-A, CTSS, HVEM, GDF-15, TIMP-1, B2M, EG-VEGF, β-IG-H3, TIMP-2, IL-6, MCP-3, PAI-1, uPAR, IGFBP-6, Dkk-1, MMP-1	SHED-CM attenuated the ALF-induced inflammation by suppressing the proinflammatory cytokine levels (IL-6, TNFα, IL-1β, and iNOS), increasing the anti-inflammatory cytokine levels (IL-10 and TGF-β1), and M2 cell markers. SHED-CM promoted hepatocyte proliferation and inhibited apoptosis. SHED-CM induced angiogenesis.	[69]
In vitro (Myelin oligodendrocyte glycoprotein-specific CD4^+^ T cells) In vivo (A mouse model of multiple sclerosis (MS))	To investigate the efficacy of SHED-CM in treating experimental autoimmune encephalomyelitis, a mouse model of MS.	n/a	In vitro: SHED-CM inhibited the proliferation of myelin oligodendrocyte glycoprotein-specific CD4^+^ T cells. In vivo: SHED-CM exhibited significantly improved disease scores, reduced demyelination, and axonal injury. SHED-CM reduced inflammatory cell infiltration and proinflammatory cytokine expression (IFN-γ, IL-17, and TNF-α) in the spinal cord.	[72]
In vitro (Schwann cells and DRG cells) In vivo (Rat model of sciatic nerve transection)	To investigate the effect of SHED-CM in the regeneration of the peripheral nerve.	NT-3, NGF, CNTF, HGF, GDNF, BDNF, VEGF	In vitro: SHED-CM significantly increased proliferation, migration, and the expression of neuron-, extracellular matrix (ECM)-, and angiogenesis-related genes in Schwann cells. SHED-CM stimulated the neuritogenesis of DRG cells and increased cell viability. SHED-CM enhanced tube formation in an angiogenesis assay. In vivo: SHED-CM promoted axon regeneration and functional recovery. SHED-CM reduced muscle atrophy.	[73]
In vitro (Cerebellar granule neurons (CGNs) isolated from newborn rats)	To evaluate the trophic actions of SHED-CM and dSHED-CM on neurite outgrowth and apoptosis in CGNs isolated from newborn rats.	n/a	SHED-CM or dSHED-CM significantly suppressed the 6-OHDA-induced apoptosis in CGNs isolated from newborn rats. Neurite outgrowth was significantly enhanced by SHED-CM and dSHED-CM.	[80]
In vitro (Glutamate-induced neurons from the cortices of C57BL/6 mice embryos) In vivo (A mouse model of Aβ-induced AD)	To investigate the therapeutic benefits of a serum-free SHED-CM in a mouse model of AD.	n/a	SHED-CM attenuated the proinflammatory (IL-1β, TNF-α, and iNOS) and induced anti-inflammatory M2-like microglia. SHED-CM improved cognitive function. SHED-CM inhibited oxidative-nitrosative stress (3-NT and iNOS) in the cerebral parenchyma. SHED-CM promoted the expression of multiple neurotrophic factors (BDNF, NGF, and IGF-1). SHED-CM suppressed glutamate-induced neuronal death.	[79]
In vivo (A mouse model of I/R)	To investigate the impact of SHED-CM on myocardial injury in a mouse model of I/R.	VEGF, IGF-1, HGF, bFGF, SDF-1, EGF, SCF	SHED-CM reduced the size of myocardial infarct, inhibited myocyte apoptosis, and suppressed inflammatory cytokine levels (IL-6, IL-1β, and TNFα).	[68]
In vivo (A rat model of SCI)	To investigate the effect of SHED-CM in a rat model of SCI.	MCP-1, ED-Siglec-9	SHED-CM improved functional recovery after SCI. SHED-CM suppressed expressions of proinflammatory cytokines (IL-1β, TNF-α, and iNOS) and induced anti-inflammatory M2-like macrophages.	[70]
In vivo (A mouse model of streptozocin-induced diabetes)	To investigate the effect of factors secreted by SHED on β-cell function and survival.	n/a	SHED-CM suppressed inflammatory chronic response of macrophage. SHED-CM promoted lung regeneration. SHED-CM increased insulin secretion, β-cell proliferation, and reduced apoptosis.	[67]
In vivo (A mouse model of BLM-induced ALI)	To investigate the effects of SHED-CM in a mouse model of BLM-induced ALI.	n/a	SHED-CM attenuated lung injury and weight loss in BLM-treated mice and improved their survival rate. SHED-CM attenuated the BLM-induced proinflammatory response and promoted the induction of anti-inflammatory M2-like lung macrophage. SHED-CM suppressed the BLM-induced tissue damage and inhibited the expression of α-SMA. SHED-CM promoted the M2 differentiation of bone marrow-derived macrophages in vitro.	[71]
In vivo (Rat model of pMCAO)	To investigate the effects of SHED-CM in a rat model of pMCAO.	n/a	SHED-CM improved motor function recovery. SHED-CM increased the expression of doublecortin (DCX), neurofilament, neuronal nuclei, and rat endothelial cell antigen in the peri-infarct area. SHED-CM induced the migration of NPCs from the subventricular zone to the peri-infarct area.	[66]
In vivo (A mouse model of perinatal HI-induced brain injury)	To investigate the effects of SHED-CM for the treatment of neonatal HI brain injury.	n/a	SHED-CM exhibited significant reductions in apoptosis and tissue loss. SHED-CM improved neurological functions.	[25]

## Data Availability

Not applicable.
